# Sugarcane bagasse: a biomass sufficiently applied for improving global energy, environment and economic sustainability

**DOI:** 10.1186/s40643-021-00440-z

**Published:** 2021-09-15

**Authors:** E. O. Ajala, J. O. Ighalo, M. A. Ajala, A. G. Adeniyi, A. M. Ayanshola

**Affiliations:** 1 Department of Chemical Engineering, University of Ilorin, Ilorin, Nigeria; 2 Department of Chemical Engineering, Nnamdi Azikiwe University, Awka, Nigeria; 3 Department of Water Resources and Environmental Engineering, University of Ilorin, Ilorin, Nigeria; 4 Unilorin Sugar Research Institute, University of Ilorin, Ilorin, Nigeria

**Keywords:** Sugarcane bagasse, Pre-treatment, Hydrolysis, Bioenergy, Bioprocessing

## Abstract

Sugarcane (*Saccharum officinarum*) bagasse (SCB) is a biomass of agricultural waste obtained from sugarcane processing that has been found in abundance globally. Due to its abundance in nature, researchers have been harnessing this biomass for numerous applications such as in energy and environmental sustainability. However, before it could be optimally utilised, it has to be pre-treated using available methods. Different pre-treatment methods were reviewed for SCB, both alkaline and alkali–acid process reveal efficient and successful approaches for obtaining higher glucose production from hydrolysis. Procedures for hydrolysis were evaluated, and results indicate that pre-treated SCB was susceptible to acid and enzymatic hydrolysis as > 80% glucose yield was obtained in both cases. The SCB could achieve a bio-ethanol (a biofuel) yield of > 0.2 g/g at optimal conditions and xylitol (a bio-product) yield at > 0.4 g/g in most cases. Thermochemical processing of SCB also gave excellent biofuel yields. The plethora of products obtained in this regard have been catalogued and elucidated extensively. As found in this study, the SCB could be used in diverse applications such as adsorbent, ion exchange resin, briquettes, ceramics, concrete, cement and polymer composites. Consequently, the SCB is a biomass with great potential to meet global energy demand and encourage environmental sustainability.

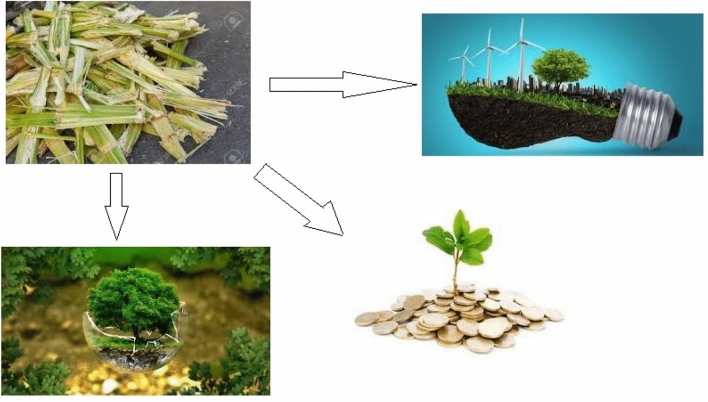

## Highlights

- Sugarcane bagasse (SCB) has been identified as a biomass that is abundantly available and can be harnessed for various applications.
- To optimally utilise SCB for its numerous applications, pre-treatment and hydrolysis are important processes.
- Various biofuels such as bio-ethanol, bio-methane, bio-hydrogen and bio-butanol have been successfully produced using SCB as a feedstock.
- Apart from being a source of energy, SCB is a sustainable feedstock for the productions of adsorbent, briquette, ceramic, concrete and composite.
- The SCB is a biomass that can be sufficiently applied for improving global energy, environment and economic sustainability.

## Introduction

Energy security and environmental conservation issues are likely to remain two of the major long-term challenges facing human existence globally (Sheikhdavoodi et al. 2015). Meanwhile, lignocellulose biomass such as sugarcane bagasse (SCB), corn stover, cereal straw, and forest woody residue (e.g., birch, spruce, eucalyptus) are substances with a high energy content that can assuage the impending energy crisis (Yin 2011; Ajala et al. 2020). They are organic materials obtained from biological sources, mostly plants biomass which is the most abundant global source of renewable materials and their annual global production has been estimated to be 1010 MT (Ajala et al. 2020). The SCB is one of these residues that are in abundance globally, which has the key to solving the global energy problem and environmental concern (Scaramucci et al. 2006).

**Fig. 1:**
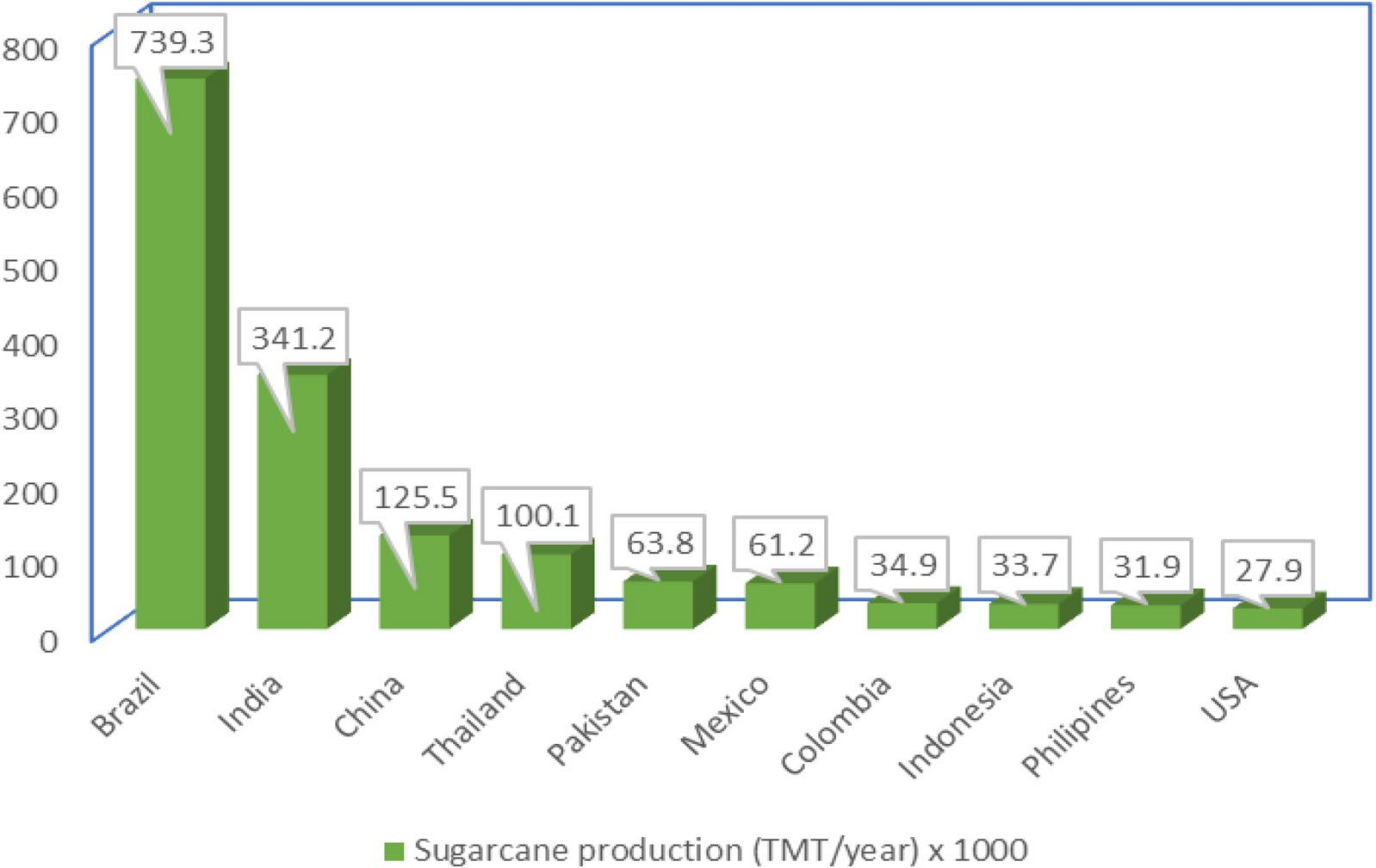
Top 10 producers of sugarcane globally (Khoo et al. 2018)

The annual production of sugarcane globally is about 1***.***6 billion tons and this generates about 279 million metric tons of SCB (Chandel et al. 2012). The global outlook for sugarcane production shows that Brazil is currently the largest producer at about 7,39,300 metric tons per year followed by India, China, Thailand, Pakistan, Mexico, Colombia, Indonesia, Philippines, and the United States (Khoo et al. 2018), as shown in Fig. 1. This indicates that the processing of this high quantity of sugarcane would unintentionally contribute to the production of a large amount of waste. The SCB from the magnitude of the sugarcane is huge which invariably poses a serious environmental concern if not attended to, hence the need for this study.

**Fig. 2:**
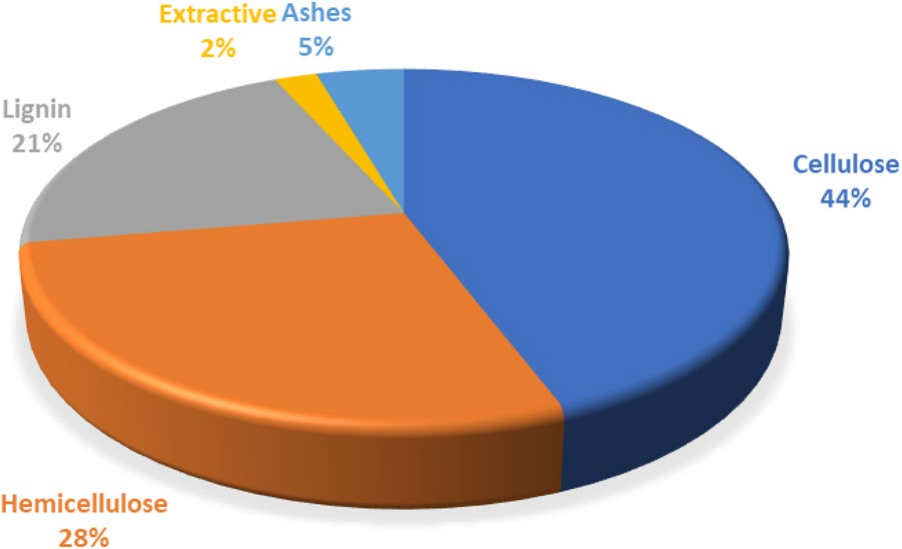
Typical chemical composition of SCB (Karp et al. 2013)

The SCB is a potential feedstock for numerous applications due to its chemical composition, as shown in Fig. 2. It is typically rich in cellulose (44%) and hemicellulose (28%), lignin (21%), ashes (5%) and extractive (2%) (Karp et al. 2013). More extensive information on chemical composition and morphology are available in the literature (Sanjuan et al. 2001; Maryana et al. 2014).

**Fig. 3:**
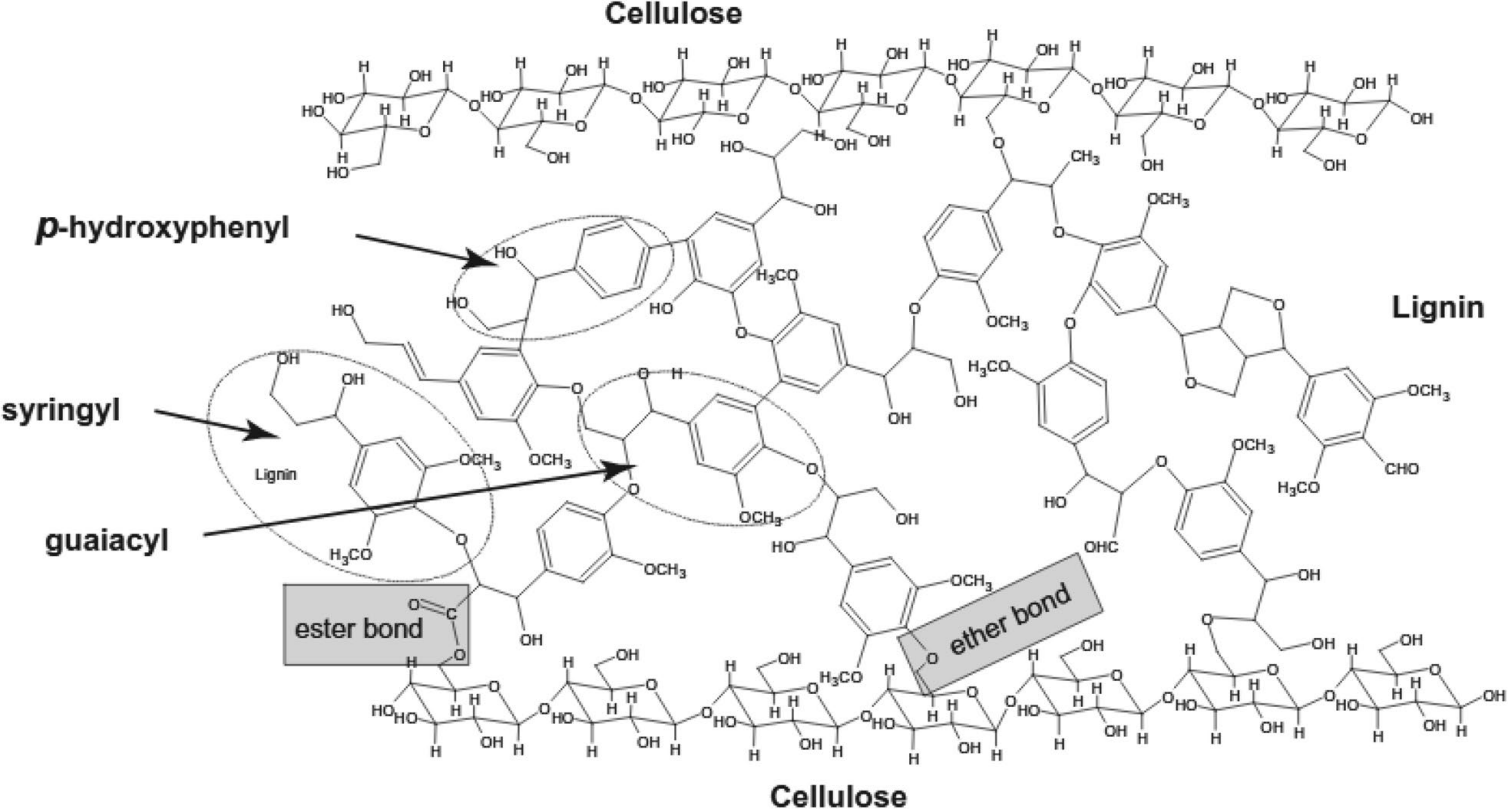
Chemical linkages between lignin and cellulose (Maryana et al. 2014)

The cellulose, hemicellulose, lignin, and small amounts of extractives and mineral salts of SCB are bonded together, physically and chemically with linkages between lignin and cell wall polysaccharides, as shown in Fig. 3.

This prevents the easy breakdown of the complex compound into simple sugars (Sun et al. 2003). This affects the rate of the de-lignification reaction and the quality of the final products. Hence, the need for pre-treatment methods that are rapid, non-destructive, and simple to isolate lignin from the cell walls of SCB (Sun et al. 2003). The methods of pre-treatment would fractionalise the SCB into its main components with high quality which is necessary to allow this renewable feedstock to be transformed into value-added products. The goal is to disrupt the complex of cellulose–hemicellulose–lignin, an important technological phase in the bio-refining of lignocellulosic materials (De Moraes Rocha et al. 2015). The pre-treatment is therefore essential for transforming SCB into high-quality fermentable sugars. Since the crystallinity of cellulose, degree of polymerisation, moisture content, surface area and lignin content are barriers to hydrolysis, which can be overcome by pre-treatment (Karp et al. 2013).

Furthermore, the SCB can be used for the production of bricks (Faria et al. 2012), ceramics (Souza et al. 2011), cement additive (Andreão et al. 2019), concrete (Payá et al. 2002), reinforcements in polymer composites (Khoo et al. 2018), biogas (Nosratpour et al. 2018), bio-ethanol (Antunes et al. 2018), bio-hydrogen (Manish and Banerjee 2008), bio-jet fuel (Diederichs et al. 2016) and adsorbents (Fideles et al. 2018). It is also used as a feedstock in pyrolysis, gasification, steam reforming, combustion, biochemical and chemical processes for the production of other valuable products. Due to the multiplicity of applications of SCB, it is important to review these uses and see the extent of work done in these areas over the years. This is an extensive review of SCB to support the fact that it is a biomass with potential for energy and environmental sustainability.

This study aimed to review various preparation techniques for optimum use of SCB for biofuel and biochemical development. The study also evaluated the important impact of various pre-treatment approaches on SCB. Few of the SCB applications that could boost the global energy outlook and maintain the environment have been considered in this work. An extensive google search of scholarly articles has been undertaken by considering publications of over 2 decades for an in-depth review on the subject of this study.

## Preparation strategies

For the SCB to be suitable as a feedstock for its numerous applications, preparation and purification to modify its physical and chemical properties are of necessity. For instance, before the SCB is used for the production of biofuels and other biochemicals, de-lignification is the initial step to be taken, followed by pre-treatment and hydrolysis. The de-lignification is necessary to make the SCB more susceptible to enzyme attack. In some processes, lignin is first broken down and removed by special enzymes or chemicals (such as alkali), before further steps are taken to convert the remaining part of the SCB to bio-ethanol (Niju and Swathika 2019).

Lignin peroxidase, manganese peroxidase and laccase are the major enzymes for de-lignification and can be produced by white-rot fungi (Malik et al. 2021). Asgher et al. (2013) undertook a comparison of both enzymatic and alkali de-lignification of SCB. Findings from the study revealed that alkali de-lignification is a better technique for maximum ethanol production. Liu et al. (2006) delignified SCB by the use of 6% sodium chloride solution at pH 3.8–4 and 75 °C for 2 h. The process was then followed by sonication for cellulose production. Rezende et al. (2011) characterised delignified SCB by chemical and morphological techniques. It was observed that the morphological changes due to de-lignification led to the improvement of enzymatic digestibility of the biomass.

The pre-treatment process also has great effects on the hemicellulose, cellulose, and lignin fraction (Antunes et al. 2018). Hemicellulose is a highly branched polymer that consists of pentose (xylose and arabinose) and hexose (glucose, mannose, and galactose) sugars (Ajala et al. 2020). However, the choice of pre-treatment method counts on the effective de-lignification and hemicellulose removal as it also has economic benefits, saves time, reduces sugar levels and causes less environmental pollution (Sabiha-Hanim et al. 2018). The general aim of pre-treatment is to boost feedstock tolerance for further processes, increase the efficiency of hydrolysis, improve overall product yield, eliminate inhibitory compounds and sterilise feedstock (Lucas et al. 2021). Therefore, for the efficient use of SCB as a biomass sufficient to boost global energy, it is important to evaluate the best pre-treatment method, which is economically viable and meets the required quality as a feedstock for industrial purposes. The use of the alkaline solution is one of the pre-treatment methods which has been experimentally proven to improve enzyme susceptibility of SCB (Aiello et al. 1996). This pre-treatment method modifies the lignin–carbohydrate complex in the feedstock by effectively interrupting the ester bonds that co-exist between lignin and hemicellulose (Cao and Aita 2013).

Maryana et al. (2014) conducted an excellent study to optimise the alkaline pre-treatment of SCB using sodium hydroxide (NaOH). Nosratpour et al. (2018) evaluated the use of various concentrations of sodium sulphite (Na_2_SO_3_), sodium carbonate (Na_2_CO_3_) and sodium acetate (CH_3_COONa) as the source of alkaline environment for the pre-treatment of SCB for bio-ethanol and bio-methane productions. The highest biogas and bio-methane productions were obtained from the SCB pre-treated with 0.5 M Na_2_CO_3_ at 140 °C. Several chemical compounds have been used for the provision of an alkaline environment in the pre-treatment process, as shown in Table 1.

**Table 1:** Chemical compounds utilised for alkaline pre-treatment

Compound	Source(s)
Sodium hydroxide (NaOH)	Maryana et al. (2014)
Ammonia (NH_3_)	Krishnan et al. (2010)
Sodium sulphite (Na_2_SO_3_)	Nosratpour et al. (2018)
Sodium carbonate (Na_2_CO_3_)	Nosratpour et al. (2018)
Sodium acetate (CH_3_COONa)	Nosratpour et al. (2018)
Calcium hydroxide (Ca(OH)_2_)	Rabelo et al. (2011)
Hydrogen peroxide (H_2_O_2_)	Rabelo et al. (2011)
Potassium hydroxide (KOH)	Bian et al. (2013)

Another pre-treatment technique in the literature is a hybrid of the acid/alkali–acid method which has been reported for the SCB. Compared to the other pre-treatment methods, acid/alkali–acid was found more effective as it is mostly used for biomass pre-treatment (Zhu et al. 2016). Teixeira et al. (1999) studied the alkali–acid pre-treatment of SCB using NaOH–peracetic acid at various concentrations to obtain a high yield of reducing sugar for bio-ethanol production. Xu et al. (2006) utilised mild alkali (1 M NaOH) and acidic 1,4-dioxane as a pre-treatment of SCB and shown to be effective. Zhao et al. (2009) made use of an alkali–peracetic acid process by first treating the SCB with 10% NaOH (at 90 °C for 1.5 h) and further de-lignifying by 10% peracetic acid (at 75 °C for 2.5 h) to obtain a reducing sugar yield of 92.04% after enzymatic hydrolysis.

The alkali–acid technique could also be assisted with a microwave energy process for the effective removal of lignin in the SCB (Binod et al. 2012). Several other pre-treatment methods have been reported in the literature for modification of SCB for efficient hydrolysis. These methods include steam explosion (Pitarelo et al. 2016), liquid hot water (LHW) (Wang et al. 2018), organosolv (Novo et al. 2011) and ionic liquid (Zhang et al. 2012).

Worthy of note is that all the aforementioned pre-treatment procedures can be combined for optimum glucose recovery from the SCB. The alkaline pre-treatment using dilute NaOH was combined with a microwave energy-assisted process for successful pre-treatment of SCB (Zhu et al. 2016). While the steam explosion was also accompanied by auto-hydrolysis (Dekker and Wallis 1983) or alkali de-lignification (Albuquerque Wanderley et al. 2013). The pre-treatment with LHW can also be used in conjunction with other reactives such as aqueous ammonia (Yu et al. 2013) and dilute alkali (Gao et al. 2013).

## Production of biofuels and bioproducts using SCB as a feedstock

For the SCB to be suitable for the production of biofuels and biochemical products, it has to be hydrolysed after the pre-treatment, otherwise, its conversion by bioprocesses such as fermentation would not be achievable. This is due to the polymeric sugars in the SCB that must be broken down into simpler units so that microbial activity can take place. The term ‘Hydrolysis’ simply means breaking down complex substances with the aid of water in the presence of a catalyst and this case of SCB, the catalyst could either be an enzyme or an inorganic chemical substance (Ajala et al. 2020).

Enzyme-catalysed hydrolysis is a biochemical process of breaking down a complex sugar into simple sugars by enzymatic action before fermentation. This is necessary to make the glucose in the complex sugar susceptible to microorganisms. The enzyme that breaks cellulose is known as cellulase which in most cases are commercial cellulases such as *Spezym, Novozym, and viscozyme.* However, culture enzymes can also be employed. The aim of enzymatic hydrolysis (and hydrolysis in general) is to break down cellulose and hemicellulose into hexose and pentose sugars (Dekker and Wallis 1983) which serve as appropriate feedstocks for the fermentation process (Pramanik et al. 2021). Cao and Aita (2013) utilised commercial enzymes (*Spezym CP* and *Novozym* 188) for the hydrolysis of SCB before fermentation by *Saccharomyces cerevisiae*. Krishnan et al. (2010) also utilised commercial enzymes (*Spezym CP* and *Novozym* 188) for the hydrolysis of SCB. However, several cultured enzymes have been reported for hydrolysis of biomass which includes *Cellulomonas flavigena* (Velmurugan and Muthukumar 2012), *Trichoderma reesei* (Rabelo et al. 2008), *Penicillium janthinellum* (Adsul et al. 2007), *Penicillium echinulatum* (Camassola and Dillon 2007) and a host of others (Maeda et al. 2011). Velmurugan and Muthukumar (2012) studied the enzymatic hydrolysis of SCB by cellulase in an ultra-sound energy-assisted process. It was shown that applying low-intensity ultrasonic energy to the process enhanced enzyme-release and intensified enzyme-catalysed reactions. Therefore, the sonication step can improve the enzymatic hydrolysis procedure (Campos et al. 2013). As shown in Table 2, enzymatic hydrolysis of SCB at a temperature range of 40–50 °C, acidic medium (4.8–6.0), moderate agitation speed and 72 h of hydrolysis yielded an excellent concentration of glucose and other hydrolysates.

**Table 2:** Different pre-treatment methods and their process conditions for enzymatic hydrolysis of SCB

Pre-treatment	Temp (^o^C)	Time (h)	Agitation (rpm)	pH	Maximum glucose yield (%)	Source
Steam	40	72	300	–	85	Carrasco et al. (2010)
Steam	50	–	150	4.8	92–94	Ewanick and Bura (2011)
Alkaline	45	72	120	4.8	-	Nosratpour et al. (2018)
Alkaline	50	–	100	4.8	87.5	Rabelo et al. (2008)
Alkaline	50	72	–	4.8	87	Antunes et al. (2018)
Alkaline	40	6	–	6.0	91.28^a, b^	Velmurugan and Muthukumar (2012)
Alkaline	45	26	150	4.8	92.11^b^	Velmurugan and Muthukumar (2012)
Steam and alkali	50	96	150	4.5	–	Adsul et al. (2007)
Organosolv	50	24	150	4.8	0.285 (g/g)	Mesa et al. (2010)
Steam	45	72	100	4.8	89.2^c^	Silva et al. (2011)
Steam	50	72	150	5.0	86	Da Silva et al. (2016)
LHW	50	72	90	4.5	0.432 (g/g)	Wang et al. (2018)
Steam	50	48	130	5.0	–	Carvalho et al. (2018)
Steam	45	96	150	4.8	–	Pitarelo et al. (2016)

^a^Yield of ethanol from final fermentation, ^b^ultrasound-assisted process, ^c^cellulose conversion

Acid hydrolysis is another method of obtaining glucose syrup from SCB which involves the use of a dilute solution of an inorganic acid to break down the complex sugar in biomass into simple sugars. The inorganic acids such as sulphuric acid, nitric acid (Rodrıguez-Chong et al. 2004) and phosphoric acid (Gámez et al. 2006) have been utilised for acid hydrolysis of SCB. Canilha et al. (2010) utilised a 2% w/v H_2_SO_4_ solution in the hydrolysis of SCB. The process was done with the acid solution and SCB in a sealed vessel heated to 150 °C for 30 min. Roberto et al. (1991) also performed an acid hydrolysis process in combination with a steam explosion pre-treatment by impregnating a 35 mM H_2_SO_4_ solution in SCB for 16 h before the subsequent steam explosion at 190 °C for 5 min.

De Moraes Rocha et al. (2011) studied the dilute acid hydrolysis of SCB by a combined sulphuric and acetic acid solution and obtained a 90% optimum conversion of the hemicellulosic hydrolysate by the 1%, w/v H_2_SO_4_ + 1%, w/v CH_3_COOH solution. Velmurugan and Muthukumar (2011) evaluated acid hydrolysis pre-treatment, but with a novel sono-assisted technique.

Ultra-sonic energy was pulsed into the system using a cycle control system and revealed that the optimal conditions for the process are 2% w/v H_2_SO_4,_ 45 min and 20:1 liquid–solid ratio. From Table 3, it was observed that some processes that utilise acid hydrolysis did not carry out any other first stage pre-treatment. This is because the acid process is capable of converting the SCB to reducing sugars in a one-pot synthesis by effectively pre-treat whilst carrying out the hydrolysis function.

**Table 3:** Summary of process conditions for acid hydrolysis

Pre-treatment	Concentration	Temp (^o^C)	Time (mins)	Maximum conversion/yield (%)	Source
	2% w/v H_2_SO_4_	150	30	30.0^a^	Canilha et al. (2010)
Steam	35 mM H_2_SO_4_	35	960	35.0^a^	Roberto et al. (1991)
	1% w/v H_2_SO_4_ + 1% w/v CH_3_COOH	190	10	90.9	De Moraes Rocha et al. (2011)
	1.8% w/v H_2_SO_4_	95	360	38.0^a^	Van Zyl et al. (1988)
Acid	2% w/v H_2_SO_4_ (ultra-sound assisted)	50	45	92.81^a^	Velmurugan and Muthukumar (2011)
–	1% v/v H_2_SO_4_	121	40	–	Borges and Pereira (2011)
–	100 mg H_2_SO_4_ per g SCB	121	10	–	Rodrigues et al. (2001)
–	Dil. H_2_SO_4_ (microwave-assisted)	180	30	–	Chen et al. (2012)

^a^Optimum yield of ethanol from fermentation

Therefore, successful conversion of SCB to reducing sugars is germane for the biomass utilisation to biofuels and biochemical products, and acid hydrolysis was reported to be preferred over the enzymatic, as it is a faster process and is highly efficient (Ajala et al. 2020).

### Biofuels

The economic viability of second-generation feedstock (corn cob, cassava peel and SCB) for biofuels production depends on their availability and process techniques, to obtain different types of biofuel (Akhabue et al. 2019). The foregoing subsections show the different techniques that could be deployed for bioprocessing of the SCB to produce respective biofuel.

#### Bio-methane (biogas)

The major constituent of biogas is methane as such can be called bio-methane, a renewable natural gas, which is a product of anaerobic digestion of biomass (SCB) (Sołowski et al. 2020). Badshah et al. (2012) evaluated the potential of SCB as a feedstock for the production of bio-methane and compare the effect of pre-treatment on the process, to determine the best route to producing the bio-methane from the SCB. Their study concluded that the acid pre-treatment is preferred above enzymatic pre-treatment to attain optimum yield of bio-methane (Ling et al. 2021). It was also observed that the maximum methane production rate from SCB is achieved after 18 days, using inoculum in the form of sludge from an anaerobic digestion factory. Rabelo et al. (2011) also studied the production of bio-methane from the pre-treated and enzymatically hydrolysed SCB which was carried out in a bioreactor at 35 °C in the presence of a buff solution, macro-elements, inoculum and oligo-elements. Upon nitrogen degasification, 165–168 L_N_ of methane/kg of biogas was obtained after 36 days.

#### Bio-ethanol

Renewability, cost-effectiveness, and environmental friendliness are the major advantages of biofuel that makes it a potential option for fossil fuels replacement (Ajala et al. 2015). Bio-ethanol is one of the examples of biofuel that can be produced from the fermentation of reducing sugars obtained from SCB by *Saccharomyces cerevisiae*. Different sources of *Saccharomyces cerevisiae* (yeast) could be used for the fermentation of hydrolysate from SCB to produce bio-ethanol (Velasco et al. 2021). Canilha et al. (2010) studied the production of bio-ethanol from SCB hydrolysate using *Pichia stipites* and obtained yield in the range of 0.2–0.3 g/g. Cao and Aita (2013) studied the production of bio-ethanol using *Saccharomyces cerevisiae* and obtained a yield of 0.2 g/g of SCB at 30 °C, 48 h incubation time and pH of 4.8. Dias et al. (2009) performed a simulation and thermal integration analysis of a conventional sugar distillery integrated bio-ethanol production with organosolv pre-treatment and dilute acid hydrolysis of SCB. The research was able to underline the value of integrating a hydrolysis plant into the power and energy optimisation sugar processing facilities. Apart from *Saccharomyces cerevisiae*, Geddes et al. (2011) reported that *Escherichia coli* is another microorganism that could be used to ferment SCB for bio-ethanol production at a temperature of 37 °C and a pH of 6.5. In their study, a maximum bio-ethanol yield of 0.21 g/g was gotten from the process which is comparatively good when compared to those of other studies, as shown in Table 4. According to Roberto et al. (1991), *Candida utilis*, *Pichia stipitis*, *Candida tropicalis* and *Pichia tannophilus* are other microorganisms that could be employed in the production of bio-ethanol. Table 4 presents a summary of the microorganisms, process conditions and bio-ethanol yields that were reported in the literature for the fermentation process of SCB.

**Table 4:** Summary of bio-ethanol production from SCB

Hydrolysis type	Microorganism	Fermentation conditions	Ethanol yield (*Y*_*P*/*S*_)	Source
Enzymatic hydrolysis	*Scheffersomyces shehatae*	30 °C, 48 h and pH of 5.5	0.34 g/g	Antunes et al. (2018)
Acid hydrolysis	*Pichia stipites*	30 °C, 48 h and pH of 5.5	0.30 g/g	Canilha et al. (2010)
Enzymatic hydrolysis	*Saccharomyces cerevisiae*	30 °C, 48 h and pH of 4.8	0.20 g/g	Cao and Aita (2013)
Enzymatic hydrolysis (SSF)	*Saccharomyces cerevisiae*	37 °C, 32 h and pH of 5.5	–	Ewanick and Bura (2011)
Enzymatic hydrolysis (L + SScF)	*Escherichia coli*	37 °C, 240 h and pH of 6.5	0.21 g/g	Geddes et al. (2011)
Acid hydrolysis	*Pichia stipites*	30 °C, 24–96 h and pH of 5.0	0.35 g/g	Roberto et al. (1991)
Acid hydrolysis	*Pichia stipites*	27 °C, 72 h and pH of 6.5	0.42 g/g	Van Zyl et al. (1988)
Acid hydrolysis (ultra-sound assisted)	*Saccharomyces cerevisiae*	30 °C, 72 h and pH of 5.0	0.36 g/g	Velmurugan and Muthukumar (2011)
Enzymatic hydrolysis (ultra-sound assisted)	*Zymomonas mobilis*	30 °C, 48 h and pH of 5.7	91.22%	Velmurugan and Muthukumar (2012)
Enzymatic hydrolysis	*Saccharomyces cerevisiae*	30 °C and 24 h	92.8%	Mesa et al. (2010)
Enzymatic hydrolysis	*Saccharomyces cerevisiae*	35 °C, 150 rpm, 34 h and pH of 4.8	–	Pitarelo et al. (2016)

#### Other bio-fuels

Some other biofuels that can be produced from the hydrolysate of SCB are bio-hydrogen and bio-butanol. Hydrogen fuel has been given great attention as an energy transmitter due to its high energy yield, lightest weight and non-release of toxic gas or carbon dioxide during the combustion process (Yue et al. 2021). The use of bio-hydrogen as a biofuel has unrivalled advantages over bio-methane such as good heating value and non-release of greenhouse gases after combustion (An et al. 2020). Recent findings revealed that bio-butanol is an improved fuel than ethanol because of its superior features, therefore, could be a potential replacement to gasoline or a better additive fuel (Prasad 2020). The amalgamation of bio-butanol with diesel and gasoline could also help towards minimising greenhouse gas emissions (Fonseca et al. 2021). The production of bio-hydrogen from renewable sources via anaerobic fermentation has been deemed as a technically possible way and has drawn more attention (Zacharia et al. 2020). It has been reported that the production of bio-butanol from hydrolysates of starch or lignocellulosic feedstock is feasible through the fermentation process (Veza et al. 2021). Fangkum and Reungsang (2011) studied the production of bio-hydrogen from SCB by utilising an inoculum rich in microorganisms from cattle dung. The hydrogen-producing bacterium in the inoculum was *Clostridium pasteurianum* and yielded maximum hydrogen of 0.84 mol H_2_/mol total sugar at a pH of 6.5. Mariano et al. (2013) investigated the technical and economic aspects of bio-butanol production from SCB in a first-generation biorefinery. It was observed that the process was more energy-intensive and has a greater volume of associated stillage. The claims by the aforementioned authors justify that the SCB is a sustainable feedstock for bio-ethanol production.

### Bioproducts

The bioprocessing of SCB is a very popular method of producing numerous biochemical substances for different applications. Several types of microorganisms like fungi (Kewalramani et al. 1988) and bacteria (Chávez-Gómez et al. 2003) are utilised to digest the SCB substrate with the desired product being the metabolic wastes of these organisms. The production of biochemical substances from SCB is important for more profitability and the ultimate achievement of economic sustainability. The availability of SCB makes it a cheaper alternative to other feedstock, an indication that the biochemicals can be produced at a lower cost. A number of bioproducts produced from SCB are thus identified and discussed based on findings in literature.

#### Xylitol

Xylitol is a sweetener used in food and pharmaceutical industries, and studies have shown that with the appropriate type of enzyme, it can be obtained from SCB substrate. Carvalho et al (2005) produced xylitol from SCB hydrolysate using *Candida guilliermondii* immobilised in calcium alginate to obtain an optimum yield of 0.81 g/g of biomass at 30 °C, 144 h, 300 rpm and pH of 6. Gurgel et al. (1995) also produced xylitol from acid-hydrolysed SCB using *Candida guilliermondii*. The study was focused on examining the best means of clarifying the fermentation broth and recovering xylitol by activation carbon treatment, ion exchange treatment then crystallisation. Martín et al. (2007) utilised an adapted strain of *Saccharomyces cerevisiae* for the production of xylitol from SCB hydrolysate in the presence of inhibitory compounds to obtain a maximum yield of 0.38 g/g biomass. A more extensive summary of xylitol production under various process conditions and microorganisms is given in Table 5.

**Table 5:** Summary of xylitol production from SCB

Method of hydrolysis	Microorganism	Process conditions	Xylitol yield (*Y*_*P*/*S*_)	Reference
Acid hydrolysis	*Candida guilliermondii*	30 °C, 144 h, 300 rpm at a pH of 6	0.62 g/g	Carvalho et al. (2005)
Acid hydrolysis	*Candida spp.*	30 °C, 60–160 h, 160 rpm	–	Chen et al. (2006)
Acid hydrolysis	*Candida guilliermondii*	30 °C, 70 h and 300 rpm	–	Gurgel et al. (1995)
-	*Saccharomyces cerevisiae*	30 °C and 48 h	0.38 g/g	Martín et al. (2007)
Steam explosion acid hydrolysis	*Debaryomyces hansenii*	40 °C, 5–10 days, 180 rpm at a pH of 6	0.69 g/g	Prakash et al. (2011)
Steam explosion acid hydrolysis	*Candida guilliermondii*	30 °C, 130 h and 200 rpm	0.48 g/g	Roberto et al. (1991)
Acid hydrolysis	*Candida guilliermondii*	30 °C, 48 h and 300 rpm and pH of 5.5	0.64 g/g	Rodrigues et al. (2003)
Acid hydrolysis	*Candida guilliermondii*	30 °C, 48 h and 200 rpm	0.54 g/g	Rodrigues et al. (2003)
–	*Candida guilliermondii*	30 °C, 24 h and 200 rpm	0.65 g/g	Santos et al. (2008)

#### Organic acids

Recently, agricultural residues, mainly lignocellulosic materials (molasses, whey, corn straw, corn cob, alfalfa fibres and waste wood) are being used as carbon sources for organic acid production. These wastes were evaluated as cheap substrates for the cost-effective fermentation of organic acid production as they would not compete with food and feed.

Therefore, exploring other cheap substrates that are renewable and environment-friendly for organic acid production are of great concern and interest to researchers. So, the use of less expensive carbon sources such as SCB instead of petroleum or natural gas to synthesise organic acid is cost-effective and advantageous as aforementioned (Ajala et al. 2021).

Studies have also shown that with the appropriate type of enzyme, organic acids such as lactic, succinic, citric and ferulic acids can be obtained from SCB substrate. Adsul et al (2007) produced lactic acid from SCB fermentation using *Lactobacillus delbrueckii*. They obtained a lactic acid yield of 0.83 g/g from a simultaneous saccharification and fermentation (SSF) process. Borges and Pereira (2011) produced succinic acid from acid-hydrolysed SCB using *Actinobacillus succinogenes* and optimised the process using response surface methodology. They obtained an optimum succinic acid yield of 0.43 g/g at 37 °C, 24 h at a pH of 7.

Kumar et al. (2003) produced citric acid from SCB substrate using *Aspergillus niger* in a solid-state fermentation process. The optimal levels of factor parameters were 75% moisture content, 31.8 g sugar/100 g dry solid, 4% w/v methanol and particles of the size between 1.2 and 1.6 mm. The optimal reported yield in the work was about 20 g/100 g dry solid. Ou et al. (2007) prepared ferulic acid from SCB by alkaline hydrolysis. The process was done with 0.5 M NaOH (with the addition of NaHSO_3_ to prevent ferulic oxidation) at 50 °C, 150 rpm and 4 h. The same research team (Ou et al. 2009), prepared coumaric acid from SCB also by alkaline hydrolysis using the same methodology. A summary of organic acids production from SCB is presented in Table 6.

**Table 6:** Summary of organic acid production from SCB

Organic acid	Method of hydrolysis	Microorganism	Process conditions	Acid yield (*Y*_*P*/*S*_)	Reference
Succinic	Acid hydrolysis	*Actinobacillus succinogenes*	37 °C, 24 h at a pH of 7	0.43 g/g	Borges and Pereira (2011)
Lactic	Enzymatic hydrolysis	*Lactobacillus delbrueckii*	42 °C, 72 h at a pH of 6	0.83 g/g	Adsul et al. (2007)
Citric	–	*Aspergillus niger*	30 °C for 9 days	0.2 g/g	Kumar et al. (2003)

#### Xylooligosaccharides

Xylooligosaccharides can be produced from xylan (derived from SCB hydrolysis) by some enzymes (Jayapal et al. 2013). It should be noted that this process is not a fermentation process but a hydrolysis process. Bian et al. (2013) examined the structural features of xylooligosaccharides derived from the action of *Pichia stipites* on SCB derived xylan and obtained the maximum conversion of 31.8% at 12 h. The hydrolysate consisted mainly of xylobiose, xylotriose, xylotetraose, xylopentose and xylohexose. Brienzo et al. (2010) produced xylooligosaccharides from SCB using *Thermoascus aurantiacus*. The maximum conversion of 37.1% was obtained at optimal conditions shown in Table 7. Besides the above reported enzymatic hydrolysis process,  a auto-hydrolysis process has also been utilised in the production of xylooligosaccharides (Zhang et al. 2021).

**Table 7:** Summary of oligosaccharides from SCB

Pre-treatment	Microorganism	Process conditions	Percentage conversion	Reference
Alkali	*Pichia stipites*	50 °C, 150 rpm, 12 h at a pH of 5.4	31.8	Bian et al. (2013)
Alkali	*Thermoascus aurantiacus*	50 °C, 150 rpm, 96 h at a pH of 5	37.1	Brienzo et al. (2010)
Steam	Auto-hydrolysis	5 N H_2_SO_4_ sol, 180 °C for 45 min	28	Fernandez et al. (2018)

#### Enzymes

The SCB has been used in numerous studies as a substrate for the production of enzymes, as presented in Table 8. Camassola and Dillon (2007) produced cellulase and hemicellulase using a solid-state fermentation process and *Penicillium echinulatum* enzyme. Cordova et al. (1998) produced lipase using a solid-state fermentation process in the presence of *Rhizomucor pusillus* and *Rhizopus rizopodiformis* enzymes as catalysts. Cunha et al. (2012) produced cellulase from SCB using *Aspergillus niger* in a sequential solid-state and submerged cultivation process. Gottschalk et al. (2010) studied the synergistic activity of *Trichoderma reesei* and *Aspergillus awamori* in the production of cellulase, xylanase, β-glucosidase and ferulic acid esterase. Although SCB possesses additional hemicellulose in contrast to paper sludge, this could be more easily reached than enzymatic hydrolysis. This is due to its lower lignin proportion which could elucidate more sugar discharged in paper sludge (Almeida Scarcella et al. 2021). Gutierrez-Correa and Tengerdy (1997) produced cellulase and β-glucosidase using *Trichoderma reesei* and *Aspergillus phoenicis* respectively, in a mixed and single-culture solid substrate fermentation process.

**Table 8:** List of enzymes produced using SCB as the substrate

Enzyme	Microorganism	Reference
Cellulase	*Penicillium echinulatum*	Camassola and Dillon (2007)
Hemicellulase	*Penicillium echinulatum*	Camassola and Dillon (2007)
Lipase	*Rhizomucor pusillus*	Cordova et al. (1998)
Lipase	*Rhizopus rizopodiformis*	
Cellulase	*Aspergillus niger*	Cunha et al. 2012) and De Souza et al. (2011)
Hemicellulase	*Aspergillus niger*	De Souza et al. (2011)
Cellulase	*Trichoderma reesei, Aspergillus awamori*	Gottschalk et al. (2010)
Xylanase	*Trichoderma reesei, Aspergillus awamori*	
β-glucosidase	*Trichoderma reesei, Aspergillus awamori*	
Ferulic acid esterase	*Trichoderma reesei, Aspergillus awamori*	
Cellulase	*Trichoderma reesei*	Gutierrez-Correa and Tengerdy (1997)
β-Glucosidase	*Aspergillus phoenicis*	
Cellulase	*Trichoderma reesei, Aspergillus niger*	Gutierrez-Correa et al. (1999)
Endoglucanase	*Trichoderma reesei, Aspergillus niger*	
β-Glucosidase	*Trichoderma reesei, Aspergillus niger*	
Xylanase	*Aspergillus fumigatus*	Lamounier et al. (2018)
β-Glucosidase	*Aspergillus fumigatus*	
Cellulase	*Ganoderma lucidum*	Manavalan et al. (2012)
Protease	*Ganoderma lucidum*	
Inulinase	*Kluyveromyces marxianus*	Mazutti et al. (2006)
Cellulase	*Trichoderma reesei*	Muthuvelayudham and Viruthagiri (2006)
Manganese peroxidase	*Phanerochaete chrysosporium*	Mohammadi and Nasernejad (2009)
α-Amylase	*Bacillus subtilis*	Rajagopalan and Krishnan (2008)
Xylanase	*Trichoderma harzianum*	Rezende et al. (2002)

Lamounier et al. (2018) used *Aspergillus fumigatus* in the saccharification of SCB extract to produce xylanase and β-glucosidase. Manavalan et al. (2012) used *Ganoderma lucidum* on SCB for the production of proteases and cellulase. Mazutti et al. (2006) produced *Inulinase* from the solid-state fermentation of SCB using *Kluyveromyces marxianus*. Mohammadi and Nasernejad (2009) utilised manganese peroxidase synthesised by *Phanerochaete chrysosporium* for the enzymatic degradation of anthracene. Table 8 gives a more exhaustive listing of enzymes produced with SCB as a substrate.

#### Other bioproducts

Poly-3-hydroxybutyrate can be obtained by the action of *Burkholderia cepacia* and *Burkholderia sacchari* on SCB (Da Silva et al. 2004). Polymer yields of 0.39 g/g and 0.29 g/g were obtained from *B. cepacia* and *B. sacchari* respectively. The SCB has been converted by alkaline and enzymatic approaches to obtain xylan of 53% (w/w) and 22% (w/w), respectively (Sporck et al. 2017). Although alkaline gave a higher yield of xylan, the enzymatic produced the xylan with the lowest contamination of lignin and glucan components. Tsigie et al. (2011) utilised SCB substrate to cultivate *Yarrowia lypolytica* for the production of lipids and obtained a maximum lipid yield of 6.68 g/L when peptone served as the nitrogen source. Cerqueira et al. (2007) produced and optimised cellulose acetate from SCB cellulose which can be used in coatings, membranes and cigar filters. In a similar process, Shaikh et al. (2009) considered the novel use of residual hemicellulose as a plasticiser in the cellulose acetate production process with positive results obtained.Chareonlimkun et al. (2010) studied the production of furfural and 5-hydroxymethylfurfural via a hot compressed water process catalysed by oxides of transition metals. The hemicellulose of SCB has been used as a precursor for the production of xylose monomers and oligomers (Jacobsen and Wyman 2002).

The SCB has also been reportedly used for the production of nano-cellulose via a high-pressure homogenisation process (Li et al. 2012). Carboxyl methyl hemicellulose (CMH) has been prepared from SCB hemicellulose by a process known as carboxymethylation using sodium mono-chloroacetate and sodium hydroxide in ethanol/water medium (Ren et al. 2008).

Da Silva (2013) depolymerised industrial organosolv lignin and traditionally extracted the lignin from SCB in the presence of an anthraquinone acid catalyst. The study was able to show the substitution of formaldehyde by glutaraldehyde (a dialdehyde that can be obtained from natural sources) by reacting the lignin with glutaraldehyde and studied as phenolic-type resins for thermosets.

## Thermochemical processing

A variety of technologies have been employed to convert biomass into valuable forms of energy (Umenweke et al. 2021). The type of conversion technology is influenced by some factors such as the type and quantity of biomass, and also the form of energy required (Prasad 2020). The SCB can be processed thermo-chemically to give value-added products that would invariably help in the achievement of energy, environmental and economic sustainability. Thermochemical processes involve very high temperatures (> 200 °C) and other process conditions which include combustion, pyrolysis and gasification (Adelodun et al. 2020). Combustion is the ignition of the biomass into flames, usually done to utilise its heating value for in-house heating, cooking or energy generation. Pyrolysis is the heating up of the biomass in a de-oxygenated environment to give bio-oil, biochar and biogas (Ighalo and Adeniyi 2020). The gasification process is of two types which are air gasification and steam gasification (commonly known as steam reforming); each of the processes mainly produces gases (Igwegbe et al. 2021; Ramos et al. 2018). The differences between both are that the former involves a constant inlet stream of air and the latter, a stream of steam (Adeniyi et al. 2019). The former yields producer gas while the latter yields synthesis gas. The process of converting the SCB to more useful products by different thermochemical processes are further reviewed in this section.

### Gasification (air gasification)

**Fig. 4:**
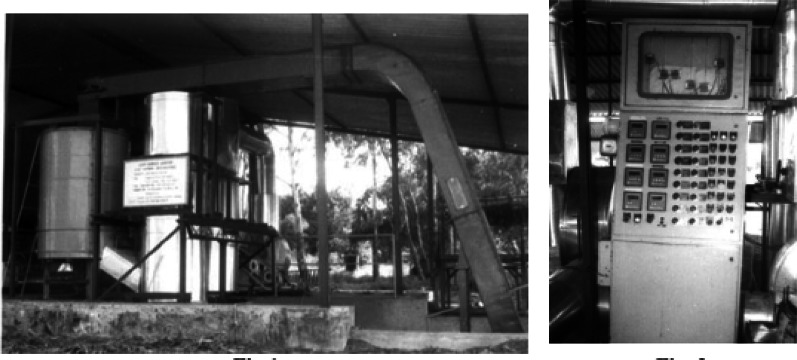
The SCB gasifier designed by Jorapur and Rajvanshi (1997)

One of the earliest designs of a gasifier specifically for SCB was by Jorapur and Rajvanshi (1997). The system included a reactor, a gas conditioning system, a biomass feeding system and an instrumentation and control system, as shown in Fig. 4. The reactor was of a downdraft configuration with a throat-less and open top. The rated thermal output of the commercial system was about 1080 MJ/h, though the operational output did not exceed 684 MJ/h. The economic analysis by Jorapur and Rajvanshi (1997) was also able to buttress that the system is profitable only if the biomass is sourced within a 30-km radius.

Predating the studies of Jorapur and Rajvanshi (1997), Gómez et al. (1999) conducted preliminary tests with SCB gasification using a fluidised bed reactor in 1996. The study pinpoints the key issues related to gasifier design at that particular time which include biomass characteristics issues that arose in the feeding system (such as clogging and bridging) and conduction of the test with an air factor greater than 0.22 was not permissible. There were major heat losses from the system as it was uninsulated, however, other more successful tests have been conducted with a cyclone gasifier (Gabra et al. 2001).

Pellegrini and de Oliveira Jr (2007) conducted an exergy analysis of SCB gasification via minimisation of Gibbs free energy model. It was deduced that the moisture content of the biomass is a major hindrance, as it was responsible for the increased destruction of exergy inside the gasification system. However, a pre-drying process to < 30% moisture was recommended for optimal gasification of SCB. Arteaga-Pérez et al. (2013) utilised a quasi-equilibrium model for their exergy analysis. Based on the exergy and energy efficiency, they put forth that the best operating temperature for SCB gasification is 1023 K and that exergy destruction within the system is about 75–80% of total losses.

**Fig. 5:**
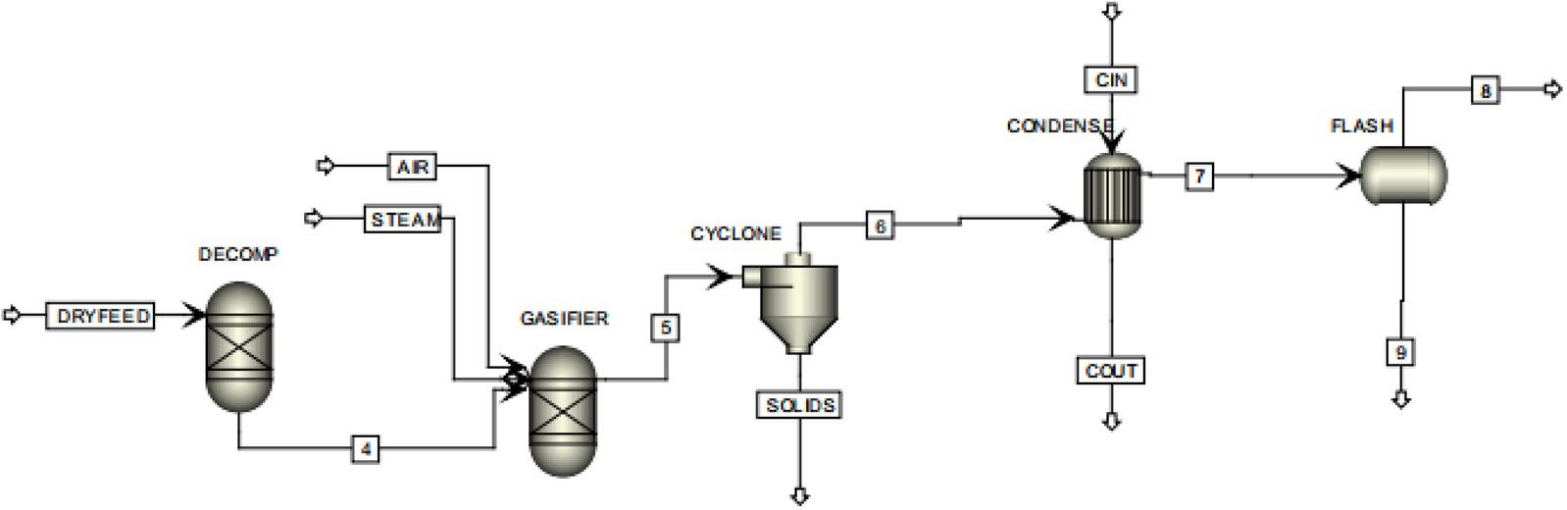
SCB gasification model on ASPEN Plus (Mavukwana et al. 2013)

Mavukwana, et al. (2013) developed and validated a simulation model on ASPEN plus for the gasification of SCB, as shown in Fig. 5. The model did not involve tar forming reactions as they are the product of non-equilibrium reactions. The gasifier itself was modelled by an RGIBBS block while the SCB biomass was broken by an RYIELD block. The simulation results were consistent with those from open literature as observed by the authors. A similar test has also been conducted with an older version of the software (Dellepiane et al. 2003).

### Steam reforming (steam gasification)

Several studies on the steam reforming of SCB have been conducted over the years. Erlich et al. (2006) in their study observed that the SCB is less reactive in the steam reforming system than wood chips as smaller pellets give less char due to a lesser decrease in the reactive volume. Waheed and Williams (2013) evaluated the potential of SCB as a feedstock in a two-stage pyrolysis steam reforming system. The optimum yield of 25.41 mmol hydrogen gas per gram of SCB was obtained at 950 °C with a dolomite catalyst of 10 wt.%. The study, however, revealed that rice husk is a better feedstock for the steam reforming process than the SCB. Osada et al. (2012) studied the steam reforming of SCB with titania-supported ruthenium (Ru) catalyst and compared it with the non-catalysed steam reforming process. The Ru-catalysed process gave a hydrogen gas yield of 3.22 mmol/g and selectivity of 14.4% whilst the non-catalysed process was 0.48 mmol/g and 9.9%, respectively. A recent study by Sheikhdavoodi et al. (2015) reported a steam reforming of SCB in a supercritical water process under potassium oxide as a catalyst to obtain optimal hydrogen yield at 75.6 mol/kg at 800 °C. However, the study revealed that the gas heating value and gasification efficiency of the alkali-catalysed process was not as good as those of other catalysts like nickel and potassium carbonate. Kruesi et al. (2013) utilised a solar-driven system in the steam reforming of SCB in a novel reactor. The study of exergy and energy showed that such a solar-driven system is energetically more advantageous than the traditional auto-thermal system. It was further discovered that the syngas produced is of superior quality (heating value) to those provided by more traditional systems. Adeniyi et al. (2019) modelled the in-line steam reforming of SCB and revealed an optimal thermodynamic condition of 600–700 °C, 1 atm and 10 kg/kg of steam-to-feed. It can be concluded that the SCB is a suitable feedstock for steam gasification if processed under appropriate conditions.

### Pyrolysis

The SCB has also been studied as a feedstock in the pyrolysis process to obtain various grades of products such as biochar, bio-gas and bio-oil. Zandersons et al. (1999) revealed the potential of SCB as a precursor for the production of biochar in a two-stage process (heating up and pyrolysis). It was concluded that SCB is a good feedstock for the production of biochar as it yielded 35% (w/w). On the other hand, the synthesis gas from SCB pyrolysis has been evaluated and considered for fuel cell applications (Al-Arni et al. 2010). The synthesis gas obtained (about 40% optimum yield) was shown to be suitable for electricity generation via fuel cells. Asadullah et al. (2007) pyrolyzed SCB to produce bio-oil and biochar as a by-product. They obtained an optimum oil yield of 66 wt.% bio-oil at 500 °C. The biochar yield was maximum at the lower temperature of 300 °C while biogas yield was maximum at the higher temperature of 700 °C. Carrier et al. (2011) undertook a comparison of slow and vacuum pyrolysis of SCB utilising response surface methodology to optimise both processes. They were able to establish the optimal conditions for maximising biochar yield, bio-oil yield, oil heating value and biochar surface area. Darmstadt et al. (2001) have earlier used SCB in a vacuum pyrolysis study by investigating the effect of petroleum residue as an additive in the pyrolysis system. They observed that co-pyrolysis yielded biochar with a small surface area. Erlich et al. (2006) also show that the size of the SCB pellets for pyrolysis has a significant effect on the product yield as small pellets (higher density) led to a high biochar yield and small shrinkage during the process. Garcìa-Pèrez et al. (2002) showed that SCB vacuum pyrolysis will give a high oil yield in a lab-scale setup than on a pilot scale and vice-versa for biochar yield. Waheed and Williams (2013) undertook a study on combined pyrolysis and steam reforming system for different feedstocks including the SCB. They revealed that the SCB will give the highest oil yield among rice husk and wheat straw under similar experimental conditions. 

Ounas et al. (2011) proceeded to carry out a stand-alone SCB pyrolysis process as opposed to the earlier combined SCB-petroleum residues co-pyrolysis. The thermal degradation patterns and activation energies of the different constituents of the biomass were elucidated. The thermogravimetric analysis clearly shows that the SCB start degradation at a temperature of 746 °C. Two peaks were observed from the analysis; the first peak was the initial mass-loss associated with hemicellulose pyrolysis which occurred between 811 and 816 °C; whereas, cellulose pyrolysis occurred at a higher temperature of between 873 and 880 °C, which was responsible for the second peak. The olive residue and SCB mostly devolatilised around 746–946 °C, with a total volatile yield of about 70–75% and the char in the final residue was about 19–26%. Also, the apparent activation energies in the 10–40% conversion range were reported to have a value of 153–162 kJ mol^−1^ and 168–180 kJ mol^−1^ and in the 50–80% conversion range, this value is 204–215 kJ mol^−1^ and 231–240 kJ mol^−1^ for olive residue and SCB, respectively. This further corroborates that the SCB can produce biochar that has activation energy as much or more than other cellulosic materials, especially olive residue. Adeniyi et al. (2019) modelled the pyrolysis of SCB in a thermodynamic predictions study and revealed that up to 63% oil yield can be obtained at 500 °C. The predictions also revealed that the oil was composed of hydrocarbons of different lengths, aromatic compounds and pyrolytic water (8.1%). The yield of SCB pyrolysis under different conditions is summarised in Table 9.

**Table 9:** Summary of product yield from SCB pyrolysis

Heating rate (^o^C/min)	Temp (^o^C)	Liquid (wt%)	Char (wt%)	Gas (wt%) [losses]	Source
60	700	19.67	22.67	48.72 [8.95]	Al-Arni et al. (2010)
50	500	66.1	24.9	9.0	Asadullah et al. (2007)
12	501	43.0	16	41^e^	Carrier et al. (2011)^a^
21.3	420	43.0	32.6	24.4^e^	Carrier et al. (2011)^b^
12	500	62.0	19.4	17.6	Darmstadt et al. (2001)
16.7	500	54.6	–	–	Drummond and Drummond (1996)
12	500	34.4	19.4	46.2^e^	Garcìa-Pèrez et al. (2002)^c^
12	500	30.1	25.7	44.2^e^	Garcìa-Pèrez et al. (2002)^d^
20	950	54.25	20.25	22.53 [2.97]	Waheed and Williams (2013)
200	500	75	5	20^e^	Tsai et al. (2006)
62.9	532	23	18	59	Kuan et al. (2013^) f^

^a^Slow pyrolysis, ^b^vacuum pyrolysis, ^c^laboratory-scale setup, ^d^pilot-scale setup, ^e^estimated by difference, ^f^non-catalysed process

### Other thermochemical processes

Combustion is the most common and the oldest way of transforming fossil fuels and biomass to valuable thermal energy. This process is used for basic cooking to more multifaceted ultra-high-pressure boilers to produce electricity (Peres et al. 2021).

Most households in the hamlet still choose to practice open fire for cooking (Athira et al. 2021). The Tropik Wood Fiji and three operational sugar mills Limited employ burning of hog fuel and bagasse, respectively in high-pressure boilers to produce electricity (Prasad 2020). The briquetting process compresses large volume loose and low-density biomass into small volume compact lumps and high density (Julia and Sarwar Khan 2021). It is a densification method that enhances the handling characteristics, calorific value and cost of transportation. Agricultural remains such as SCB are usually used for briquetting and they can be completed with or without a binder (Silveira Rossi et al. 2021). Briquetting process is cheaper, has higher practical thermal value, has no sulphur content and is non-polluting, has low ash content, is renewable, ideally sized for uniform and complete combustion, eco-friendly and economical (Zhang et al. 2021).

Ahmad et al. (2018) considered an integrated process for the thermo-catalytic reforming of SCB in a lab-scale reactor. At the experimental optimal conditions, they obtained 57 wt.% gaseous products, 23.5 wt.% biochar, 4 wt.% bio-oil and 15.5 wt.% aqueous phase. The gaseous product was 37% hydrogen and possessed a heating rate of 16.40 MJ/kg and the bio-oil obtained possessed a relatively low water (2.6 wt.%) and oxygen (10.2 wt.%) content. Chen et al. (2012) studied the hydrothermal carbonisation of SCB via a wet torrefaction process with water, spiked with sulphuric acid and heated in a microwave-assisted process. The sulphuric acid doping was shown to have a positive effect on the process. Ramajo-Escalera et al. (2006) modelled the kinetics of SCB dehydration and combustion process; the SCB combustion flames had low luminosity, nearly transparent and spherical, however, the char combustion was bright and short-lived. The thermal analysis and de-volatilisation kinetics of SCB in inert and non-inert environments have also been investigated (Munir et al. 2009). In all these, we can surmise that the combustion of SCB is a rapid process generating a relatively good amount of thermal energy.

## Adsorbents

The SCB is an excellent precursor for the development of adsorbents for the removal of pollutants from aqueous solutions. The utilisation of biomass as an adsorbent has been expressed as an effective and cheaper technology for the removal of the adsorbate of various forms from wastewater (Adelodun et al. 2021). The SCB has been used as an adsorbent for the removal of heavy metals (Khoo et al. 2018), dyes (Fideles et al. 2018) and motor oil (Sun et al. 2004). The adsorbents can be in the form of biosorbent (Brandão et al. 2010), activated carbon (Xia et al. 1998) or ion exchange resins (Laszlo 1996). The preparation of SCB biosorbent is usually by washing, soaking in a chemical reagent to improve the surface properties, rinsing, drying, grinding and sieving (Eletta et al. 2020). The activated carbon is prepared by chemical or steam activation and carbonising in a furnace at very high temperatures (Adeniyi and Ighalo 2019).

Girgis et al. (1994) in their study have revealed that phosphoric acid is a better chemical activation agent for the carbonisation of SCB than other chemicals such as sulphuric acid, hydrochloric acid and nitric acid. An essential approach is the steam explosion, which serves as a physical treatment that augments the biomass surface area and as a result can enhances other treatments, such as enzymatic hydrolysis (Lucas et al. 2020). Abdullah et al. (2005) observed that sulphuric acid treatment of SCB is better than the formaldehyde treatment, for the removal of methylene red from an aqueous solution. They found out that the optimum pH for the biosorption process was 9 in every case. At 25 °C and pH 5 the biomass had an experimental adsorption capacity of 2 mg/g and the process was exothermic and spontaneous. Ali et al. (2012) utilised raw ground SCB as a sorbent for the removal of oil from oil–water mixtures. Their studies showed an extra 10 g/g of SCB sorption ability, thus revealing its positive potential to be used in this regard. Krishnan et al. (2011) studied the adsorption of Pb (II) unto unmodified SCB and SCB activated carbon (AC). The specific surface area of the AC from SCB was 536.5 m^2^/g compared to raw SCB which was 146.785 m^2^/g and commercial AC which was 452.635 m^2^/g. The SCB AC also showed a greater adsorption capacity for nickel (which was 140.85 mg/g) than raw SCB (which was 73.56 mg/g) and commercial AC (which was 111.11 mg/g). Brandão et al. (2010) examined the biosorption of petroleum hydrocarbons onto SCB biosorbent. The biosorbent was able to remove 99% gasoline and 90% n-heptane from an aqueous solution showing its potential to serve as a treatment agent for hydrocarbon polluted waters. Carvalho et al. (2011) conducted a study to evaluate the potential of SCB as a sorbent for phosphate and were able to show that chemical modification of the biosorbent by FeCl_2_ (referred to as doping with Fe^2+^) can improve the adsorption capacity by about 42%. Cronje et al. (2011) optimised the adsorption of hexavalent chromium by SCB activated carbon using response surface methodology; central composite design. The optimums were 6.85 g/L dosages, 40 °C temperature, 77.5 mg/l initial metal concentration and pH of 8.58. 

Other researchers have also reported the use of modified SCB for the adsorption of hexavalent chromium (Garg et al. 2009), copper, cobalt and nickel (Xavier et al. 2018). The study achieved excellent removal of the aforementioned heavy metals from wastewater using modified SCB. Da Silva et al. (2011) utilised an elaborate preparation technique that includes NaOH, mono-chloroacetic acid, ethanol and FeCl_3_.6H_2_O, to modify SCB for the biosorption process of brilliant red from aqueous solution. Besides the excellent adsorption capacity obtained as shown in Table 10, their studies revealed the best fits for the Avrami fractional kinetic model and Sips equilibrium model. Inyang et al. (2011) were able to show that activated carbon can be obtained from anaerobically digested SCB which can have 2 times more sorption capacity for lead than commercial activated carbon. The developed adsorbent had 20 times more sorption capacity than the raw SCB. Qureshi et al. (2008) utilised SCB as a precursor for the development of activated carbon aimed at sugar decolourisation. Their study revealed that it showed better decolourising property than commercial carbon. The SCB has also been reported for use in a mixture with compost and granular activated carbon (GAC) for the removal of benzene, toluene, ethylbenzene and o-xylene (BTEX) from the air stream in a packed bio-filter (Mathur et al. 2007). The adsorbent showed a maximum removal efficiency of 99% for all compounds studied. Sene et al. (2002) also examined SCB packaging as a bio-filter for benzene in gaseous streams. Their results were not so positive as the elimination capacity was lower than for other standard bio-filters. In a similar air pollution study, Pantoja Filho et al. (2010) were interested in hydrogen sulphide gas-phase sorption unto SCB bio-filters. It was revealed that SCB bio-filters is an excellent long-term bio-filter for hydrogen sulphide after displaying a 100% removal efficiency (after 2 days).

**Table 10:** Summary of SCB application as adsorbents

Nature of bagasse	Modification/activation reagent	Adsorbate	Monolayer adsorption capacity (mg/g)	Source
Biosorbent	Sulphuric acid	Methylene red	–	Abdullah et al. (2005)
	Formaldehyde	Methylene red	–	
	Untreated	Methylene red	–	
Biosorbent	Untreated	Ni^2+^	2.234, 2^a^	Alomá et al. (2012)
Activated carbon (AC)	Steam	Ni^2+^	140.85	Krishnan et al. (2011)
Biosorbent (pith)	Untreated	Ni^2+^	73.56	
Biosorbent	Untreated	Gasoline	8.36 mL/g	Brandão et al. (2010)
Biosorbent	Untreated	n-Heptane	2.78 mL/g	
Biosorbent	NaOH	Phosphate	67.5	Carvalho et al. (2011)
Biosorbent	NaOH, FeCl_2_	Phosphate	152	
AC	ZnCl_2_	Cr^6+^	–	Cronje et al. (2011)
Biosorbent (lignin)	NaOH, mono-chloroacetic acid, ethanol, FeCl_3_.6H_2_O	Brilliant red 2BE	60.3	Da Silva et al. (2011)
Biosorbent	Trimellitic acid, pyridine, dimethyl-acetamide	Auramine-O	2.492 mmol/g	Fideles et al. (2018)
Biosorbent	Trimellitic acid, pyridine, dimethyl-acetamide	Safranin-T	1.23 mmol/g	
Biosorbent	Succinic acid	Cr^6+^	–	Garg et al. (2009)
Biosorbent	Succinic anhydride	Cu^2+^	185.2	Gurgel et al. (2008)
		Cd^2+^	212.8	
		Pb^2+^	416.7	
Biosorbent (mercerised)	NaOH, succinic anhydride	Cu^2+^	185.2	Gurgel et al. (2008)
		Cd^2+^	256.4	
		Pb^2+^	500.0	
Biosorbent (mercerised)	NaOH, acetone, 1,3-diisopropyl-carbordiimide, dimethyl-formamide, triethylene-tetramine	Cu^2+^	69.4	Gurgel and Gil (2009)
		Cd^2+^	106.4	
		Pb^2+^	222.2	
Biosorbent	Succinic acid, pyridine	Methylene blue	478.47	Gusmão et al. (2012)
		Gentian violet	1273.16	
Biosorbent	Ethylenediamine tetra-acetic acid (EDTA) dianhydride	Methylene blue	202.43	Gusmão et al. (2013)
		Gentian violet	327.87	
AC	Epichlorohydrin, dimethyl-formamide, trimethylamine	Nitrate	5.82	Hafshejani et al. (2016)
Biosorbent	Untreated	Basic violet 10	50.4	Ho et al. (2005)
Biosorbent	Untreated	Basic violet 1	20.6	
Biosorbent	Untreated	Basic green 4	13.9	
Biosorbent	H_2_SO_4_, NaOH	Cd^2+^	1.95 mol/kg	Homagai et al. (2010)
		Pb^2+^	1.58 mol/kg	
		Ni^2+^	2.52 mol/kg	
		Zn^2+^	2.40 mol/kg	
		Cu^2+^	2.91 mol/kg	
AC (from anaerobically digested SCB)	–	Pb^2+^	653.9 mmol/kg	Inyang et al. (2011)
AC	Steam	Chlorine	–	Jaguaribe et al. (2005)
Biosorbent (mercerised)	Ethylenediamine tetra-acetic acid (EDTA) dianhydride, pyridine, acetic anhydride	Cu^2+^	92.6	Júnior et al. (2009)
		Cd^2+^	149.0	
		Pb^2+^	333.0	
AC	ZnCl_2_	Phenol	12.33	Kalderis et al. (2008)
Biosorbent	Untreated	Hg^2+^	35.71	Khoramzadeh et al. (2013)
AC (Pith)	HCl, steam	Pb^2+^	200.0	Krishnan and Anirudhan (Krishnan and Anirudhan 2002)
		Hg^2+^	188.68	
		Cd^2+^	153.85	
		Co^2+^	128.70	
AC (pith)	Steam	Cd^2+^	24.70	Krishnan and Anirudhan (2003)
Biosorbent	Untreated	Cu^2+^	9.48	Liu et al. (2012)
Biosorbent	Untreated	Pb^2+^	6.366	Martín-Lara et al. (2010)
Biosorbent	Sulphuric acid	Pb^2+^	7.297	
Biosorbent (cellulose)	Zirconium oxychloride	Sulphate	0.4 mol/g	Mulinari and Silva (2008)
Biosorbent	Ethylenediamine tetra-acetic acid (EDTA) dianhydride, pyridine, acetic anhydride	Zn^2+^	102.25	Pereira et al. (2010)
Biosorbent (lignin)	Formic acid, NaOH, chloroacetic acid	Cd^2+^	–	Peternele et al. (1999)
		Pb^2+^	–	
Biosorbent	Untreated	Methyl red	5.66	Saad et al. (2010)
Biosorbent	Phosphoric acid	Methyl red	10.96	
Biosorbent (with sludge)	KOH, HCl, HNO_3_	Pb^2+^	135.54	Tao et al. (2015)
Biosorbent (rind)	Immobilised in Ca-alginate	Cr^3+^	296.21	Ullah et al. (2013)
		Cr^6+^	495.56	
Biosorbent (pith)	Immobilised in Ca-alginate	Cr^3+^	381.05	
		Cr^6+^	767.25	
Biosorbent (rind beads)	Immobilised in Ca-alginate	Cr^3+^	303.11	
		Cr^6+^	491.24	
Biosorbent (pith beads)	Immobilised in Ca-alginate	Cr^3+^	449.23	
		Cr^6+^	832.13	
Biosorbent	Pyromellitic dianhydride, FeCl_3_, FeSO_4_, EDTA	Pb^2+^	1.2 mmol/g	Yu et al. (2013)
		Cd^2+^	1.1 mmol/g	
Biosorbent	Untreated	Congo red	38.2	Zhang et al. (2011)
Biosorbent	Untreated	Rhodamine B	65.5	Zhang et al. (2013)
		Basic blue 9	30.7	

^a^Experimental adsorption capacity

An interesting application of SCB is also its novel use in the synthesis of zeolite nano-adsorbent which can be used as an ion-exchanger, as a catalyst and as an adsorbent (Moisés et al. 2013). These among other studies elucidated in Table 10, go a long way to prove that the SCB is an excellent material for the development of low-cost adsorbents suitable in removing heavy metals, dyes, organic solvents, hydrocarbons and many other environmental pollutants from aqueous solutions.

## Other applications of SCB

This section highlights other important uses of SCB as a potential feedstock for economic sustainability. The SCB is a potential source of flavonoids (Colombo et al. 2006), for soil amendment (Deng et al. 2016), to produce super-capacitor electrodes (Rufford et al. 2010) and for the synthesis of zeolite nano-adsorbent (Moisés et al. 2013). Also, SCB can be used for the generation of electricity via combustion in thermal power plants. Life cycle assessment has shown that the process is sustainable in terms of resources though at the cost of a negative environmental impact (Silva et al. 2014). The SCB has also being used as a substrate for the cultivation of edible mushrooms (Moda et al. 2005). It has been considered as feedstock for the preparation and purification of lignin–carbohydrate complexes (also known as commercial lignin) (Singh et al. 2005; Hoareau et al. 2004). The SCB is highly rich in carbohydrates as it can be consumed as food for human beings, feeds for the animal in numerous forms and serves as fertiliser for crop production (Pramanik et al. 2021).

## Briquettes, cements and composites

The SCB can also be used in numerous environmental civil engineering applications. It can be used for making bricks, concretes, ceramics, cement, and as re-enforcement for plastic composites (Loh et al. 2013). SCB ash is a residue obtained from the burning of SCB for heat generation in boilers and electricity generation purposes. This ash is used as cementitious material due to its pozzolanic properties (Ofuyatan et al. 2021). In this section, progress made so far in research on the use of SCB for environmental engineering are further elaborated.

### Briquettes and ceramics

Due to the high silicon oxide component of sugarcane bagasse ash (SCBA), it has been considered as an additive for ceramics and high-temperature quartz bricks. Table 11 presents some properties of these bricks and ceramics used in high-temperature applications. Souza et al. (2011) evaluated the feasibility of its use in ceramics and was observed that the addition of SCBA is advantageous due to behaviours like a non-plastic material in the bricks; thereby reducing its linear shrinkage during drying and firing. The SCBA has been considered as a potential replacement for quartz in red ceramics. It has been shown that the properties of the ceramics can be improved with up to 10 wt% addition of SCBA to form the ceramic bricks (Teixeira et al. 2008). Faria et al. (2012) also studied the use of SCB for a high-temperature application, albeit with clay as the major component of the bricks. Though advantageous, it was succinctly elucidated that the SCBA has limitations in high-temperature bricks. Major concerns were the increased water absorbance and the reduction of the mechanical properties especially above 10 wt.% SCBA. Lima et al. (2012) analysed the mechanical properties of compressed earth bricks doped with SCBA for masonry applications.

**Table 11:** Summary of properties of SCB bricks for high-temperature applications

SCB or SCBA (Wt%)	Temp (^o^C)	Linear shrinkage (%)	Water absorbance (%)	Bulk density (g/cm^3^)	Flexural strength (MPa)	Apparent porosity (%)	Reference
10	1000	2.25	–	–	10	–	Teixeira et al. (2008)
20	1000	3	22	1.7	6	14	Souza et al. (2011)
10	1000	6	22	1.65	12.5^a^	29	Phonphuak and Chindaprasirt (2018)
10	1000	2.25	22.8	1.67	1.5^b^	–	Faria et al. (2012)

^a^Compressive strength, ^b^tensile strength

In one of the more significant conclusions from the study, it was surmised that with 12wt% cement and 8wt% SCBA, the bricks are excellent and can be used in the manufacture of non-structural masonry components which proves the technical feasibility of this material.

### Cement and concrete

Payá et al. (2002) considered SCBA as a cement replacing material in the concrete industry. The major conclusion from the study was quite negative but was valid nonetheless. It was observed that the SCBA is too fine and possesses a relatively high inorganic carbon content (about 15%) to be used in concrete production. They suggested further studies if SCBA will be successfully used in cement. Sales and Lima (2010) went further to do a study on this area. It was realised that the ash cannot be referred to as a substitute for the mortars as it cannot match the binding properties of cement. It can be considered as a sand substitute as its properties are similar to those of very fine sand. It was observed that partial substitution of 20–30% with SCBA was optimum for the compressive strength of the concrete. Pereira et al. (2018) decided to examine the use of SCBA in cement mortars. Replacement in the range of 15–20% yielded the best behaviour in terms of compressive strength alone. It was also once again observed that the effect of SCBA doping on other mechanical properties is sometimes negative.

Ganesan et al. (2007) observed an optimum of 20% partial substitution. Numerous other studies have been conducted in this respect with similar conclusion (Andreão et al. 2019; Bahurudeen et al. 2014). Other studies have come in the domain of performance evaluation (Bahurudeen et al. 2015), the pozzolanic effect (Frías et al. 2011) and the filler effect (Cordeiro et al. 2009). In a recent study, SCBA application in self-compacting concrete was examined to replace cement by up to 30%. It was observed that the proportion of SCBA used did not significantly improve the mechanical properties of the concrete, except for a slight improvement in the compressive strength (Moretti et al. 2018).

### Polymer composites

Composites are structural materials consisting of two or more constituents (sometimes not soluble in each other) combined at a macroscopic level (Adeyanju et al. 2021). It consists of the reinforcing or discontinuous phase (metal, ceramic and fibre) embedded in the continuous phase or matrix or resin (Thermosets and Thermoplastics) (Ighalo et al. 2021). Bilba et al. (2003) studied the influence of the chemical composition of SCB on the properties of bagasse-cement composites. The major observation of the study was that the presence of the SCB in the composite delays the setting time and decreases the maximum hydration temperature of the setting. The tests by Cerqueira et al. (2011) showed that SCB fibres will significantly improve the mechanical properties of Polypropylene (PP)-SCB composites. The 15wt% SCB gave maximum impact and flexural strength while 10wt% SCB gave maximum tensile strength. Luz et al. (2007) also studied the mechanical behaviours of PP-SCB composites and compared compression moulding and injection moulding. They also conducted a thorough microstructural analysis of the composites. From the study, it was put forward that injection moulding under vacuum is the better preparation technique (when considering the properties of the end products). Luz et al. (2008) utilised cellulose and lignocellulose from SCB in the development of PP composites.

The study was focused on the effect of acetylation of the fibres on the mechanical and thermal properties of the end product. It was discovered that acetylation of the fibres is not an effective fibre pre-treatment process as it led to products with lesser mechanical properties. Luz et al. (2010) went on to show that PP-SCB composites were more environmentally friendly than Talc-PP composites. The former is lighter but with equivalent performance in automotive applications.

Hoareau et al. (2006) developed phenol–formaldehyde and SCB composites in the form of fibreboards. From their measurements of impact strength and water absorption, it was put forth that pressure application during the curing process is important and dilution of the lingo-phenolic pre-polymer to ensure good interaction between the fibres and the resin is important too. Paiva and Frollini (2002) established the technical feasibility of developing phenol–formaldehyde and SCB composites and buttressed that the pre-modification do not have a significant effect/improvement on the final product. Mulinari et al. (2009) prepared high-density polyethene (HDPE)-SCB composites. They observed that the modification of SCB cellulose reduced the composites elongation at the break by 15% in comparison to unmodified SCB cellulose but increased the tensile modulus by 38%. The modification was by 10% sulphuric acid, 1% sodium hydroxide then sodium chloride bleaching followed by the use of zirconium oxychloride. A similar conclusion on the general effect of such modification was also reinforced by another similar study for HDPE-SCB composites (Mulinari et al. 2010). Rodrigues et al. (2011) developed polyester (PE)-SCB resins by an esterification pre-modification of fibres. Their study revealed that chemical modification improved tensile strength by as much as 71.5% compared to PE resin alone but only by 13% for unmodified fibres. It was attributed to the fact that the pre-treatment improved the interfacial bonding ability of the fibres with the resins.

Stael et al. (2001) have also been able to show the feasibility of deriving ethylene-*co*-vinyl acetate (EVA)-SCB composites. Table 12 presents a summary of the mechanical properties of SCB in polymer composites.

**Table 12:** Mechanical properties of SCB reinforced polymer composites

Composite	SCB in composite (wt%)	Impact strength (kJ/m^2^)	Tensile strength (MPa)	Flexural strength (MPa)	Tensile modulus (MPa)	Elongation at break (%)	Source
PP-SCB	5	0.0327	22.9	34.8	1105.5	–	Cerqueira et al. (2011)
PP-SCB	10	0.0425	23.0	35.5	1027.1	–	
PP-SCB	15	0.0525	22.3	37.2	1442.5	–	
PP-SCB^a^	5	–	18.7	19.2	1001.9	3	Luz et al. (2007)
HDPE-SCB	10	–	1.54	–	897	1.74	Mulinari et al. (2009)
HDPE-SCB	10	–	15.6	–	1324.2	1.2	Mulinari et al. (2009)
PE-SCB^b^	5	–	11	–	1358.1	–	Rodrigues et al. (2011)

*PP* polypropylene, *HDPE* high-density polyethene, *P* phenolic thermoset, *PE* polyester

^a^Obtained by compression moulding, ^b^unmodified fibres

## Techno-economic analysis

Several studies have been performed over the years to determine the techno-economic viability and financial profitability of the SCB for various applications. This section appraised their finding to justify that the SCB is an economically sustainable feedstock. Cavalett et al. (2012) conducted a techno-economic analysis of the first-generation refining of SCB and reported that the return on investment is higher in autonomous plants than annexed plants. Also, the return on investment was higher in fixed plants than in flexible plants. In both fixed and versatile processes, it was found that annexed plants have a higher return investment rate (RIR) than the autonomous ones. Flexibility has been argued as a crucial index in sustaining productivity in this business sector. As global sugar demand is unlikely to exceed the demand for ethanol, this should be taken into account in the design of the plants. Dias et al. (2010) carried out an economic assessment of sugarcane ethanol production from an autonomous distillery. It has been noted that the use of the produced waste (SCB) as fuel in the power cogeneration system would significantly boost the process’s profitability. And the selling of generated excess power is another option too. Dantas et al. (2013) assessed the cost and viability of the different technical routes for SCB electricity generation. The study's key finding is that electricity generation by combustion of biomass is still the only economically feasible option at the moment. The current position still holds even though predictions were made up to the year 2030 for an improvement in the electrical generation from SCB. The other technologies are still considered to be in their infancy and early-stage growth (except for the Rankine cycle process).

Gubicza et al. (2016) carried out a techno-economic study of SCB bio-ethanol production. The mechanism involved was a liquefaction mechanism with *Escherichia coli* concurrently saccharifying and co-fermenting. The key cost contributors have been described as the feedstock price (contributing 25% annual cost of production) and the annual cost of capital (contributing 45% total cost of production). It is well understood that ethanol yield can affect the cost of production, and it has been shown in this study that it is financially appropriate to increase the concentration of enzymes (increasing enzyme cost) to boost ethanol yield. In an interesting study, Leibbrandt (2010) compared the techno-economic viability of biochemical processing to the thermochemical processing of SCB. It was observed that biological treatment with liquid hot water and acid hydrolysis pre-treatment were not energy self-sufficient, but the steam explosion pre-treatment was energy self-sufficient. Both fast and vacuum pyrolysis and Fischer–Tropsch processing were energy self-sufficient. It was observed that Fischer–Tropsch processing has the highest total investment cost followed by steam explosion pre-treatment, then fast pyrolysis and finally vacuum pyrolysis, for bio-ethanol production. The order still holds for liquid fuel production cost while the reverse order holds for the internal rate of return.

Macrelli et al. (2012) studied the economics of 2nd generation bio-ethanol production from SCB and leaves, integrated with sugar-based ethanol production processes. It was observed that the average ethanol selling price (for first- and second-generation processes) at 0.53 US$/L is economically feasible for all types of processes. Mesa et al. (2016) evaluated bio-ethanol production based on two SCB pre-treatment strategies; organosolv and enzymatic hydrolysis. Based on their economic consideration, the proposed best alternative is 15 min using an acid pulping solution without ethanol in a solid-to-liquid ratio of 1 g/5 mL; and the second step of 60 min using 45% (v/v) of ethanol and 3% of NaOH on dry fibre. The study presented a technology that can be applied at an industrial scale due to the elevated ethanol yield and low operational costs involved.

Seabra et al. (2010) considered the economics of biochemical and thermochemical processing of sugarcane residues as a side process to the main sugar refining process. They revealed that the biochemical conversion of the residues may lead to an additional 0.033 m^3^ ethanol per tonne of cane and the thermochemical conversion will lead to about 0.025 m^3^ per tonne. It was also revealed that electricity will be an important co-product for the biorefinery, especially for the biochemical conversion process. Merwe (2013) compared the energy efficiencies and economics of different process designs for bio-butanol production from sugarcane molasses. The fermentation process with *C. beijerinckii* in a fed-batch system with in situ gas stripping, followed by liquid–liquid extraction (LLE) and steam stripping distillation was the only profitable process based on prevailing economic reality. Though the process was energy efficient, it was put forward that using molasses will result in large fluctuations in the product selling price which can ultimately undermine the viability of such production process.

It has also been shown that producing bio-butanol from SCB through first-generation refining is still as profitable as the bio-ethanol process (Mariano et al. 2013). Mariano et al. (2013) were also able to show that bio-butanol production is a more profitable process in general than biogas production. In their cursory look at the bio-ethanol production from SCB, Ensinas et al. (2013) opined that the major problem of the second-generation bio-ethanol production processes is the high cost of enzymes. Jorapur and Rajvanshi (1997) revealed that the generation of energy via the gasification of SCB is a profitable venture. It was also put forward that the economics become more favourable with larger gasification systems due to the economics of scale. Based on this techno-economic appraisal of SCB, it can be concluded that it a viable feedstock for economic sustainability. This is because it can be used to produce several products at optimum profit margin with adequate return on investment.

## Conclusion

The various applications of SCB have been extensively examined in light of the need for energy and environmental sustainability. The SCB can be regarded as a sustainable feedstock for biofuels production as it was successfully used to produce bio-ethanol, bio-methane, bio-hydrogen, and bio-butanol. Bio-products such as xylitol, organic acids, xylooligosaccharides and enzymes that were produced from the SCB justify its economic importance. The diversity of SCB applications was even more amazing as the material has also found applications as adsorbent, ion exchange resin, briquettes, ceramics, concrete, cement, and polymer composites. It can be surmised that SCB is biomass with great potential to supplement global energy demand and foster environmental and economic sustainability.

## Data Availability

Not applicable.

## Abbreviations

SCB: Sugarcane bagasse
SCBA: Sugarcane bagasse ash
LHW: Liquid hot water
HPLC: High-performance liquid chromatography
EVA: Ethylene-*co*-vinyl acetate
HDPE: High-density polyethene
LLE: Liquid–liquid extraction
PP: Polypropylene
PE: Polyester
RIR: Return investment rate
